# A Retrospective Analysis of the Safety and Efficacy of Opioid-free Anesthesia versus Opioid Anesthesia for General Cesarean Section

**DOI:** 10.7759/cureus.5725

**Published:** 2019-09-22

**Authors:** Garrett Enten, Mina A Shenouda, David Samuels, Naomi Fowler, Maha Balouch, Enrico Camporesi

**Affiliations:** 1 Research, Tampa General Hospital, Tampa, USA; 2 Anesthesia, Tampa General Hospital, Tampa, USA; 3 Miscellaneous, University of South Florida, Tampa, USA

**Keywords:** cesarean section, anesthesia, opioid, perioperative and pain medicine

## Abstract

Introduction

While uncommon for cesarean delivery, general anesthesia may be patient requested or necessary due to maternal contraindication. Traditionally, opioids are used as a part of the general anesthetic. Because of their associated complications, it is standard to limit opioid use and fetal narcotic exposure during cesarean delivery. We conducted a retrospective study to evaluate the feasibility of multi-modal opioid-free general anesthesia for cesarean delivery.

Methods

Electronic medical records were obtained for patients receiving general anesthesia for cesarean delivery of live pregnancies through 2017 at our tertiary care facility. Post-operative pain was estimated using a 10-cm visual analogue scale and by calculating postoperative narcotic requirements in milligram morphine equivalents (MME) over three-time periods: during post-anesthesia recovery in the post-anesthesia care unit (PACU), the first 24 hrs after PACU discharge, and 24-48 hrs after PACU discharge. Apgar scores were also obtained to quantify neonatal effects of the general anesthetic.

Results

Eight of 17 patients (47.06%) received opioid-free anesthesia (OFA), and nine of 17 patients (52.94%) received anesthesia with opioids (OA). No significant difference was found between groups in terms of postoperative mean Visual Analog Scale (VAS) pain score over each time period. Similarly, no significant difference was found between groups in terms of postoperative narcotics requirement at all study points. Apgar scores were not significantly different between the two groups.

Conclusion

The OFA group displayed equivalent analgesia to the OA group in terms of self-reported VAS pain scores and postoperative MME. A larger prospective study is recommended to fully evaluate OFA for cesarean delivery.

## Introduction

Cesarean section is performed for 30% of live births, making it one of the most common surgical procedures in the United States [1]. As rates of cesarean delivery (CD) continue to rise globally, continuous improvements have been published to enhance the patient outcomes for this procedure [2]. Anesthetic-associated obstetric mortality has decreased to seventh on the list of causes for maternal mortality in the USA and remains at rates of 1-3 maternal deaths per million maternities in both the USA and UK [3,4]. General reductions in obstetric mortality were seen after 1980 and are attributed to the increase in regional anesthesia for cesarean delivery (CD), improved safety of regional anesthesia techniques, as well as algorithms and airway devices to improve safety of general anesthesia [5]. In the past few decades, the use of general anesthesia for CD has decreased dramatically. A recent hospital survey from 2005 estimates that only 0.6% of CD performed in tertiary care facilities utilize general anesthesia [6]. The rise in epidural anesthesia during labor, evolution of multi-modal pain management approaches, and the desire to avoid fetal exposure to depressant medications and to allow the mother to remain awake during delivery have been instrumental to these changes [7]. However, a general anesthetic may still be requested by patients or may be required in non-emergent CD as contraindications for regional anesthesia may arise such as abnormal maternal hemodynamics, neurological abnormalities or spinal instrumentation.

Current literature suggests that opioids are commonly prescribed during pregnancy [8]. Newborns are affected by these analgesics administered during labor and delivery. The use of opioids for labor analgesia, either neuraxial or systemic, has been linked with undesired maternal and neonatal outcomes. Current methods of decreasing fetal opioid exposure include clamping of the umbilical cord prior to maternal opioid infusion, minimizing opioids prior to delivery, and delivering the baby in a timely fashion. Nevertheless, limiting opioid use to maternal exposure can still lead to undesirable outcomes. Neuraxial morphine-related side effects include pruritus, nausea, urinary retention, and respiratory depression, although the risk for the latter is significantly lower when morphine is administered neuraxially than systemically [9, 10]. Removing opioids from the general anesthetic altogether will eliminate the risk of potential maternal and fetal opioid exposure.

In this patient series, we compared the maternal and neonatal outcomes between two groups receiving general anesthesia during CD: patients receiving opioid-free anesthesia (OFA) vs. patients receiving opioid anesthesia (OA). An ongoing hospital group wide quality improvement initiative to minimize or remove opioid for intraoperative anesthesia across all specialties allowed for the opportunity to evaluate the effects of opioids in general anesthetic for CD. Opioid-free cases were generated through patient education of our ongoing initiative and accommodation of the patients’ request to avoid opioid exposure. We hypothesized that equivalent analgesia in terms of patient reported pain and postoperative narcotics requirement can be provided utilizing a multimodal opioid-free approach. Further, we expected to see improved maternal and fetal outcomes compared to the OA group.

## Materials and methods

After obtaining approval from the University of South Florida IRB (Pro00033429, 3/6/2018), we conducted a retrospective chart review of patients undergoing CD from March 2017 to December 2017, at Tampa General Hospital (Tampa, Florida). The patients evaluated in this series specifically requested general anesthesia or were not candidates for regional anesthesia due to contraindications such as spinal instrumentation or thrombocytopenia. Patient and care provider data were extracted from the electronic medical record system using a standardized data collection form. Patients who received no pre- or intraoperative narcotics were classified in the OFA group. The review comprised 17 opioid naive patients who underwent general anesthesia for CD using the same anesthesia nursing staff and surgeons over nine months during the same period (March 2017 to December 2017). Eight patients received OFA, and nine patients received OA.

The OFA patient group received a preemptive analgesic dose of 1000 mg acetaminophen PO and 400 mg of Gabapentin PO prior to surgery. Additionally, the OFA group received a pre-op treatment dose of 20 mg famotidine PO as a prophylactic for nausea. Subsequently, both patient groups received induction with propofol, succinylcholine, and rapid sequence intubation with sevoflurane up to 1.5 MAC. In all cases, the umbilical cord was clamped promptly, and the baby delivered before any other anesthetic drug was administered to the mother. Prior to delivery but after the umbilical clamp was in place, OFA patients received ketamine 0.5 mg/kg followed by magnesium sulfate 60 mg/kg over 20-30 mins and lidocaine 1.5-2 mg /kg/hr, while OA patients received fentanyl 200-500 mcg. Post-delivery, all patients received ketorolac 15-30 mg. Magnesium sulfate was not continued post-operatively for fear of intra-uterine bleeding. As postoperative pain management was handled by the obstetric team, standard postoperative opioid pain management was resumed after surgery. Visual Analog Scale (VAS) pain scores on a 10-cm scale were obtained every 15 minutes during post-anesthesia recovery. IV hydromorphone was administered PRN to both groups during their stay in the post-anesthesia care unit (PACU). After discharge from the PACU, pain scores were taken every hour; should the patient be resting and unresponsive a VAS score of 0 was recorded. Pain management after the first 48 hours after PACU discharge consisted of PO oxycodone-acetaminophen and PO NSAIDs PRN.

Demographics, surgical characteristics, neonatal outcome metrics, and length of stay were collected. Persistent nausea was marked in patients that required further medication in addition to the standard 15 mg postoperative dose of ondansetron. Apgar scores were evaluated by placing neonates in categorical ranges 0-3, 4-6, and 7-10 where: scores between 7 and 10 indicate routine post-delivery care, scores between 4 and 6 indicate the potential need for respiratory aid, and scores under 4 may require more invasive interventions.

Postoperative analgesic requirements were measured quantitatively using acetaminophen dosage, ASAS-NSAID Equivalency Scores, and milligram morphine equivalents (MME) as calculated by posted guidelines of the CDC. Each point of measure was calculated for the duration of the procedure, PACU stay, first 24 hr after PACU discharge, and 24-48 hr after PACU discharge. All continuous endpoints were analyzed using unpaired two tailed t-tests. Fisher’s Exact tests were used to compare categorical endpoints between groups. Statistical significance was determined with a p-value < 0.05.

## Results

There was no significant difference between the two groups in terms of demographics or surgical/recovery characteristics (Table 1). Both patient groups showed negligible difference in age, body mass index, surgery duration, and estimated blood loss allowing further comparison to be drawn between group outcome measures. Pain as measured by self-reported pain scores on a 10-cm VAS pain scale and postoperative analgesic requirement in terms of acetaminophen dose, ASAS-NSAID Equivalency Scores, and MME were not significantly different (Table 2). Though not significant, a visible reduction in 24 hr acetaminophen dose and ASAS-NSAID Equivalency Scores can be visualized. Ultimately this may be attributed to standard dosing practices as a preemptive analgesic dose of 1000 mg acetaminophen was used in the OFA group. This supports the first portion of our hypothesis showing equivalent analgesia between groups. With no major complications or breakthrough pain reported for either group, these results may indicate analgesic equivalency between both pain management courses. Of note, persistent nausea requiring treatment with promethazine only occurred within the OA group (22.2% vs. 0.0%, p = 0.4706).

**Table 1:** Patient Demographics and Surgery/Recovery Characteristics BMI: Body mass index

Variable	Mean +/- SE	Mean +/- SE	p-value
	Opioid Anesthesia (OA) n = 9	Opioid-free Anesthesia (OFA) n = 8	Unpaired t-test
Age (year)	26.3 ± 1.5	29.5 ± 1.8	0.1937
BMI (Kg/m^2^)	33.7 ± 2.7	31.3 ± 1.8	0.4969
% Receiving Elective Surgery	56%	75%	0.4023
Length of Surgery (min)	68 ± 9.6	76 ± 10.3	0.6025
Estimated Blood Loss (ml)	872 ± 335	1088 ± 582	0.3573
Length of Post Anesthesia Care Unit stay (min)	146 ± 10.9	143 ± 22.6	0.9133
Time to marked Alert after Post Anesthesia Care Unit admission (mins)	73.5 ± 25.3	76.4 ± 29.2	0.9411
Persistent Nausea During Recovery (%)	22.0%	0.0%	0.4706
Hospital Length of Stay (day)	3.6 ± 0.24	3.9 ± 0.23	0.3544

**Table 2:** Comparison of Analgesic Requirements and Self-reported Pain: OA vs. OFA PACU: Post-anesthesia care unit; NSAID: Nonsteroidal anti-inflammatory drug; ASAS: Assessment of SpondyloArthritis International Society; MME: Milligram morphine equivalents.

Variable	Mean +/- SE	Mean +/- SE	p-value
	Opioid Anesthesia (OA) n = 9	Opioid-free Anesthesia (OFA) n = 8	Unpaired t-test
Mean Visual Analog Pain Score at PACU	4.3 ± 0.97	4.6 ± 0.97	0.8311
Mean Visual Analog Pain Score 24 hr	3.7 ± 0.58	3.1 ± 0.41	0.4083
Mean Visual Analog Pain Score 24 to 48 hr	3.5 ± 0.68	4.3 ± 0.65	0.4189
Mean Total 24 hr NSAID Use (Acetylsalicylic Acid Non-steroidal Anti-inflammatory Drug) ASAS NSAID Equivalency Score	97.2 ± 8.2	63.5 ± 16.7	0.0807
Mean Total 24 to 48 hr NSAID Use (Acetylsalicylic Acid Non-steroidal Anti-inflammatory Drug) ASAS NSAID Equivalency Score	84.3 ± 9.7	80.2 ± 18.0	0.8402
Mean Total 24 hr Acetaminophen Use (mg)	722.2 ± 240.9	243.8 ± 101.9	0.1008
Mean Total 24 to 48 hr Acetaminophen Use (mg)	613.9 ± 183.2	650.0 ± 212.8	0.8988
Mean Total Narcotics Use at PACU (MME)	9.2 ± 2.5	7.4 ± 2.7	0.6428
Mean Total Narcotics Use 24 hr (MME)	24.6 ± 5.8	29.5 ± 6.0	0.5712
Mean Total Narcotics Use 24 to 48 hr (MME)	6.9 ± 2.1	11.3 ± 3.9	0.3270

In terms of neonatal outcomes, while not significant due to the restrictive sample size, a higher percentage of neonates in the OA group presented with Apgar scores ranging from 4-6 at 1 and 5 minutes (Table 3). Upon review of neonatal endpoints, Apgar scores were not significantly different at 1 minute or at 5 minutes for neonates of patients receiving opioid and patients on opioid-free anesthesia. No neonates ever experienced an Apgar score ≤ 3.

**Table 3:** Chi-squared Analysis of Neonatal Outcomes: OA vs. OFA

	Opioid Anesthesia (OA) n = 9	Opioid-free Anesthesia (OFA) n = 8	p-value
Apgar at 1 min (Percent ≤ 6)	44.4%	12.5%	0.2941
Apgar at 5 min (Percent ≤ 6)	11.1%	0.0%	1.000

## Discussion

Regional anesthesia has become the standard for both elective and emergent CD. Historically, general anesthesia has been associated with several fetal complications, albeit these data are a bit dated today [11]. More recent literature has begun to overturn this notion, showing that regional anesthesia is not superior to general anesthesia in terms of major maternal or neonatal outcomes. Korkmaz et al. found no differences in the 1-minute and 5-minute Apgar scores when comparing epidural - spinal anesthesia versus general anesthesia [12]. Additionally, a 2007 Cochrane review reported that when considering neonates with Apgar scores less than 4 and 6 at 1 and 5 minutes, the proportion of infants who were delivered under general anesthesia was not significantly different from the proportion of those delivered under regional anesthesia [13]. Despite this, doctors have been slow to incorporate general anesthesia back into their tool chests as the associated risks of failed endotracheal intubation and aspiration of gastric contents remain a concern for obstetric anesthesia providers [14]. However, physicians may not have a choice. Demand increases for local anesthetics have created shortages in the past, limiting the availability of regional anesthesia techniques. General anesthesia may be an appropriate alternative if the need should arise, but it remains indicated for elective cesarean delivery cases due to maternal contraindications for regional anesthesia such as spinal instrumentations or hemodynamic instability.

Opioids are commonly used during cesarean delivery to achieve rapid induction during general anesthesia and to ensure satisfaction with pain relief postoperatively. Commonly, fentanyl patient-controlled intravenous analgesia is utilized in the post-anesthesia recovery unit, typically 25 μg every 10 to 15 minutes, with an hourly lockout of up to 100 μg [15]. However, many studies observed that patient satisfaction for labor analgesia is not affected only by reduction in pain intensity. A randomized trial noted that while pain scores reductions were greater with regional analgesia, patient satisfaction scores were not affected [16]. The epidemic use, abuse, and misuse of opioids in the United States today have resulted in significant morbidity and mortality. Opioids can provoke many postoperative side effects. Some of these side effects, including postoperative nausea and vomiting (PONV), are obvious; some are of a more subtle nature such as tolerance and dependence [17]. In this study, patients who received opioids intraoperatively were more likely to experience persistent nausea in PACU, requiring treatment with promethazine, compared to patients in the OFA group (22.2% vs. 0.0%, p = 0.4706). While not significant due to limited sample size, a tendency towards respiratory depression in the OA group was noted by a larger percentage of neonatal Apgar scores at 1 and 5 minutes. Furthermore, undesirable side effects such as constipation, urinary retention, and drowsiness may significantly impede an otherwise uneventful postoperative course [18]. While the data collected during recovery in the PACU showed that there was no difference in mean time to marked alert by nursing staff, results may be confounded by the effect of postoperative opioids ordered by the surgeon.

In 2014, Fletcher and Martinez published a meta-analysis on postoperative opioid-induced hyperalgesia [19]. They included 19 prospective studies comparing pain scores, morphine consumption, and hyperalgesia after high- vs. low-dose remifentanil or placebo in adult patients undergoing surgery. The authors concluded that high intraoperative doses of remifentanil are associated with significant increases in acute pain intensity and increased morphine use during the first 24 h after surgery. In the current study, no significant change in pain score between the two groups has been recorded. Various combinations of non-opioids have been successfully used both for intra-operative anesthetics and for post-operative analgesia. A recent example for critically ill patients utilized continuous infusions of lidocaine plus dexmedetomidine [20].

Our results from both groups suggest that opioid-free anesthesia in cesarean delivery is possible as we did not observe any significant changes regarding fetal and maternal sequelae between the two groups. However, the current study is limited by the small number of patients studied and by many confounding factors. The administration of opioid analgesics initiated during post-anesthesia recovery does blur our primary and secondary endpoint measurements due to opioid-associated side-effects, including narcotics requirement in 48 hrs after surgery, PONV, and evaluation of time to patient designated as “alert” by PACU nurses. Nevertheless, with no major complications or patient complaints, this brief series demonstrates that opioid-free anesthesia is feasible for CD and can produce comparable results to opioid anesthesia regarding procedure duration, blood loss, Apgar scores, and perioperative analgesia as measured by postoperative narcotics requirement and VAS pain scores. Further research and a prospective study on a larger group of patients undergoing OFA and OA cesarean delivery will be necessary to evaluate the feasibility of opioid-free anesthesia in cases of cesarean delivery.

## Conclusions

The OFA group displayed equivalent analgesia to the OA group in terms of self-reported VAS pain scores and postoperative MME. A larger prospective study is recommended to fully evaluate OFA for cesarean delivery.
